# Literature review and case report on the use of rezafungin for spondylodiscitis in an outpatient parenteral antimicrobial therapy (OPAT) setting

**DOI:** 10.5194/jbji-11-395-2026

**Published:** 2026-07-06

**Authors:** Alexander J. Richards, Ammara Asif, Monica Ivan, Patrick J. Lillie, Gavin Barlow, Chloe Walsh

**Affiliations:** 1 Infection Research Group, Hull University Teaching Hospitals NHS Trust, Hull, UK; 2 Hull York Medical School, University of York, York, UK

## Abstract

Bone and joint infections secondary to *Candida* species are associated with high morbidity and mortality, requiring extended courses of antifungal therapy, with parenteral medications being first-line therapy. The reported use of antifungals in outpatient parenteral antimicrobial therapy (OPAT) for deep-seated bone and joint infections is limited. We report and discuss the use of rezafungin, a novel long-acting echinocandin, in an OPAT setting for spondylodiscitis secondary to *Candida albicans*.

## 1 Introduction

Fungal spinal infections are rare, with an increasing incidence in recent years (Ganesh et al., 2015). Invasive *Candidiasis* is associated with high morbidity and mortality (Friedman and Schwartz, 2019) and due to complexity often requires extended courses of parenteral (intravenous; IV) therapy, resulting in prolonged inpatient admissions.

Echinocandins are currently the recommended first-line agents for invasive candida infections (Pappas et al., 2016; Cornely et al., 2012), although their documented use in an outpatient parenteral antimicrobial therapy (OPAT) setting is limited (Burnett et al., 2021; Gil-Navarro et al., 2020). Rezafungin is a novel second-generation echinocandin that has recently been brought to market for treatment of candidemia and invasive candidiasis (Sofjan et al., 2018).

Rezafungin is a structural modification of anidulafungin, where the C5 carbinolamine group is replaced by choline aminal ether, increasing structural stability and solubility, which results in a prolonged half-life of 130 h, permitting a once-weekly intravenous (IV) dosing regimen (Davidson et al., 2025). In vitro evidence suggests that this structural modification may also confer improved anti-biofilm properties in comparison to traditional echinocandins, which may be useful in deep-seated infections (Abduljalil et al., 2026). In a recent phase 3 trial (Thompson et al., 2022), rezafungin was found to be non-inferior to caspofungin, a once-daily IV echinocandin with a comparable safety profile, making it an attractive OPAT option.

We present the case of Ms X, a 67-year-old female, presenting with recurrent *Candida albicans* infection and multilevel spondylodiscitis at the Hull University Teaching Hospital's regional Department of Infection. The patient had significant azole intolerance, preventing oral therapy, and poor mobility, prohibiting clinically delivered once-daily OPAT antifungal administration. We then go on to summarise the reported bone and joint infection cases treated by rezafungin in the literature to date.

## 2 Clinical case

The patient initially presented in early July of 2024 with gallstone disease, leading to ileus and bowel obstruction. During this initial episode of admission, Ms X required an emergency laparotomy with an overnight postoperative intensive care unit (ICU) stay. The patient initially improved, leading to hospital discharge at day 7 of admission.

The patient returned to hospital 6 d later with worsening abdominal pain and was subsequently diagnosed with persistent small bowel obstruction with gallstone ileus, requiring further laparotomy. Post-operatively, the patient went to ICU for blood pressure support and was later transferred to a general surgical ward. The patient required a prolonged period of bowel rest and total parenteral nutrition (TPN) via central venous catheter (CVC).

Almost 2 months into this admission, the patient experienced spiking fevers and felt generally unwell; *Candida albicans* was subsequently isolated from both CVC and peripheral blood cultures. This was initially thought to be secondary to TPN-CVC infection. As there were no signs of dissemination with a normal echocardiogram, a 14 d course of IV anidulafungin was planned post line removal. However, during the initial phase of treatment, the patient was noted to have increasingly blurred vision and was subsequently diagnosed with retinitis after an ophthalmology review, with the planned course of antifungal therapy extended to 6 weeks.

Later that month, the patient was also treated for an *Escherichia coli* and *Enterococcus faecalis* bloodstream infection, again thought to be secondary to a second CVC line infection; she received IV teicoplanin and aztreonam for this, and the line was removed. The patient clinically and biochemically improved thereafter and was discharged in early November 2024.

The patient was re-admitted in December 2024 after having been off antimicrobial therapy for 6 weeks, with upper- and lower-back pain radiating to the legs and buttocks. An MRI of the spine with intravenous contrast on admission demonstrated marked inflammatory changes (high-STIR and low-T1 signal changes) in both the cervical (C3–C4) and the lumbar (L2–S1) disc spaces and vertebral bodies with moderate narrowing of the spinal canal at C3–C4 and severe narrowing at L2–L4, along with right-exit neural foraminal narrowing, in keeping with spondylodiscitis. One set of three blood cultures again isolated a fully sensitive *Candida albicans* (sensitivities – minimum inhibitory concentration – MIC mg L^−1^): anidulafungin-S – (0.0015), voriconazole-S – (0.03), fluconazole-S – (1), micafungin-S – ( <0.008 ), for which anidulafungin was started along with teicoplanin and aztreonam to cover the previous *E. coli* and *E. faecalis* bloodstream isolates. Susceptibility testing and interpretation followed the Clinical and Laboratory Standards Institute (CLSI) guidance (CLSI, 2020) as per local laboratory policy, rezafungin MIC testing was not performed, and anidulafungin was used a surrogate marker as per Winkler et al. (2025).

After discussion with the neurosurgical team, it was felt that the MRI images along with the positive blood cultures were sufficient evidence to treat for spondylodiscitis and a biopsy was not needed.

Anidulafungin was continued for 4 d before being changed to 800 mg of oral fluconazole once a day due to loss of IV access. IV antibiotics were also switched to oral ciprofloxacin and linezolid. One week later, the patient's condition worsened such that she was unable to take oral medications, and IV anidulafungin was restarted (along with IV linezolid and ceftriaxone).

Ceftriaxone and linezolid were stopped at day 14 after discussion in the infection multidisciplinary (MDT) meeting, during which it was agreed that, based on the blood culture being positive only for *Candida* spp. (with no other pathogens being grown on re-admission) and clinical assessment, the likely diagnosis was *Candida albicans* multi-level spondylodiscitis. A 6-month course of antifungals alone was agreed.

The patient continued to clinically and biochemically improve on anidulafungin. To aid movement to a complex physiotherapy rehabilitation bed, the central line was removed 34 d into treatment, and she was switched to 400 mg of oral fluconazole once a day. The patient developed profuse nausea and vomiting with raised alkaline phosphatase (peak of 391 IU L^−1^) with normal alamine transferase and bilirubin, thought to be fluconazole induced, which progressed despite switching to oral posaconazole (standard-release gastro-resistant tablets – 300 mg twice a day for 24 h loading followed by 300 mg once a day). The patient was therefore transferred back to the infection ward and recommenced IV anidulafungin.

Daily IV options were not considered a long-term solution as the local OPAT service is based on a self-administration or clinic-attendance-based infusion model with no service for home infusion delivery; the patient was unsuitable for self-administration and unable to attend daily OPAT due to a lack of transport. The patient was therefore commenced on IV rezafungin at a 400 mg loading dose followed by 200 mg once weekly, via the OPAT service.

The patient continued to improve, with normalisation of inflammatory markers (peak white cell $22.7\times 10^{9}$ L^−1^, peak c-reactive protein 315 mg L^−1^) and improved pain, and did not recrudesce 7 months after stopping rezafungin therapy. In total, she completed 6 months of IV antifungal therapy, 4.5 months of which was rezafungin. While on rezafungin, the patient reported no subjective adverse effects but did have recurrent episodes of mild serum hypokalaemia (lowest potassium 3.0 mmol L^−1^ – normal range 3.5 to 5.3 mmol L^−1^), without electrocardiogram (ECG) changes, requiring intermittent oral potassium supplementation. Episodes of hypokalaemia resolved on cessation of rezafungin. MRI of the spine with intravenous contrast 2 months post-treatment (8 months after admission MRI) showed “no new spinal lesions with stable appearance of L2–L3 disc spaces with stable narrowing of L2–L4 neural exit. Resolution of L5–S1 changes” and “moderate degenerative changes of the cervical spine C3–C4 with a possible inflammatory pannus measuring 9 mm”. The repeat MRI was discussed in the spinal MDT, and appearances agreed to be likely due to post-infective changes.

## 3 Summary of reported cases to date

We found eight reported cases of rezafungin being used to treat bone and joint infections secondary to *Candida* (Table 1; see Appendix for search strategy). These cases reported different antifungal regimens pre-rezafungin use, but all report positive clinical outcomes, with resolution of infection in six cases and with two cases receiving ongoing treatment at the time of the report (see Appendix for literature search strategy). *Candida albicans* represents a minority of the causative organisms reported in the literature with regard to the use of rezafungin, with the majority being *Nakaseomyces glabrata* (formerly *Candida glabrata*) or mixed infection.

**Table 1:** Literature review of rezafungin use in *Candida* bone and joint infection cases – L-AmB, liposomal amphotericin B.

Source	Diagnosis	Organism	Antifungal therapy (duration if known)	Outpatient or inpatient rezafungin	Surgical intervention	Rational for rezafungin	Outcome (follow-up time)
Chiurlo (2023)	Prosthetic joint infection, knee	*N. glabrata*	Anidulafungin and caspofungin Rezafungin (18 weeks)	Not stated	Nil	Not stated	Resolved (not stated)
Ponta et al. (2024)	Sacral osteomyelitis	*C. tropicalis* + *N. glabrata*	Caspofungin (3 months) Rezafungin (8 weeks)	Outpatient	Surgical debridement	Patient convenience and tissue penetration	Resolved (not stated)
Lahouati et al. (2024)	Spondylodiscitis	*N. glabrata*	Caspofungin Rezafungin (10 weeks)	Not stated	Nil	Patient convenience and azole resistance	Resolved (not stated)
Viceconte et al. (2024)	Spondylodiscitis	*C. parapsilosis*	Anidulafungin (10 d) Voriconazole + L-AmB (2 weeks) Rezafungin 26 weeks	Outpatient	Nil	OPAT, reduced susceptibility to azoles and intolerance to azoles and L-AmB	Resolved (not stated)
Kmet et al. (2025)	Intra-abdominal collection and spondylodiscitis	*C. albicans*	Caspofungin (11 d) Fluconazole (12 weeks) Fluconazole + L-AmB (15 weeks) Rezafungin (8 weeks)	Outpatient	Surgical drainage of abdominal collection	Facilitate discharge	Resolved (not stated)
Trapani et al. (2025)	Metal-work-associated spondylodiscitis	*C. albicans* + *N. glabrata*	Fluconazole Caspofungin Rezafungin (39 weeks)	Outpatient	Nil	OPAT and long-term treatment required	Improving (ongoing)
Keck et al. (2025)	Prosthetic joint infection, hip	*C. parapsilosis*	Rezafungin (12 weeks)	Outpatient	Nil	Interactions with azoles, to avoid CVC	Resolved (not stated)
Davidson et al. (2025)	Spondylodiscitis	*N. glabrata*	Anidulafungin Rezafungin (2 weeks)	Outpatient	Nil	Patient convenience, facilitate discharge and azole resistance	Improving (ongoing)

Both the CSLI and the European Committee on Antimicrobial Susceptibility Testing (EUCAST) do provide MIC breakpoints for rezafungin (CLSI, 2025; EUCAST, 2025); however this modified susceptibility-testing method is not routinely undertaken in our local laboratory. While EUCAST does not explicitly provide guidance on extrapolating susceptibility testing from other echinocandins, Winkler et al. (2025) found that anidulafungin is the best-performing surrogate marker for *Candida* spp., with the exceptions of *C. dubliniensis* and *C. auris*. This alignment between anidulafungin and rezafungin is not surprising given their related chemical lineage.

The association between the use of rezafungin as a treatment for the typically more resistant *Candida* species may be due to the wider range of suitable oral treatment options in *Candida albicans* infections and/or the increasing incidence of *non-albicans Candida* species infections (CDC, 2024) but also highlights the potential to treat such infections as outpatient cases in patients who cannot access OPAT central infusion sites daily or undertake self-administration.

The literature highlights that there is no consistent and standardised management strategy for the management of fungal bone and joint infections, with variation in practice demonstrated. Rezafungin has been administered in the OPAT setting in all but one case (Keck et al., 2025) but predominantly only after protracted periods of hospitalisation and the use of other traditional antifungals, either in combination therapy or in monotherapy.

In the reported cases, rezafungin was used as monotherapy, and there were no reported adverse events attributed to the rezafungin. In our case, the patient experienced mild hypokalaemia, which was clinically thought to be secondary to rezafungin. Hypokalaemia is listed in the medicine patient information leaflet (Cidara Therapeutics, 2025) as a “very common” side effect, affecting more than 1 in 10 people, and emphasises the need for at least weekly blood test monitoring in the outpatient setting.

The case that we present of a CVC infection with spinal seeding of infection caused by *Candida albicans* and subsequent relapse, leading to spondylodiscitis, highlights the complexity and need for a specialist MDT approach in managing deep-seated fungal bone and joint infections. These infections require long durations of often IV and antifungal therapy, sometimes in patients who do not have access to traditional daily OPAT because of mobility, patient geography, family support, or physical capability issues. Inability to access traditional daily OPAT can result in prolonged hospital admissions for IV therapy or an earlier-than-desirable switch to oral therapy, which may not be tolerated as in this case, adding to the burden of resources and bed pressures and potentially increasing the risk of hospital complications, such as healthcare-acquired infection. The high cost of branded rezafungin (no generic version currently available) may be prohibitive in some cases, particularly if discharge is not possible for other reasons. When deciding on the use of rezafungin, the approach to decision-making should therefore take into account the purchase cost, OPAT costs, and cost of available alternatives in the context of the patient's clinical circumstances.

Rezafungin in Ms X's case allowed for the continued OPAT service care of an infection that would have otherwise resulted in a further 4.5-month hospital stay due to a lack of availability of effective oral antifungal therapy. The case also adds to the growing evidence base that rezafungin, with its favourable pharmacokinetics, has the potential to be a valuable tool in the OPAT armamentarium in reducing inpatient stays whilst effectively treating candidemia and invasive candidiasis.

## Data Availability

Case data are not publicly available as the data are held on private patient information systems.

## Appendix A Description of literature review

**Databases**: PubMed and Medline
**Language**: English language
**Dates**: all reported cases
**Search terms**:
  – rezafungin
  – outpatient antimicrobial therapy (OPAT)
  – antifungal
  – novel antifungal
  – long-acting antifungal
  – long-acting echinocandin
  – discitis
  – spondylodiscitis
  – spinal infection
  – bone and joint infection
  – osteomyelitis
  – prosthetic joint infection
  – native joint infection
  – articular infection
